# ReSNNance: an HDL generation framework for spiking neural networks

**DOI:** 10.3389/fnins.2026.1958451

**Published:** 2026-10-13

**Authors:** Samuel López-Asunción, Cristina Bermúdez-Martín, Pablo Ituero, Marisa Lopez-Vallejo

**Affiliations:** 1 Information Processing and Telecommunications Center, Escuela Técnica Superior de Ingenieros de Telecomunicación, Universidad Politécnica de Madrid, Madrid, Spain; 2 Universidad Politécnica de Madrid - Indra, Madrid, Spain

**Keywords:** automatic VHDL generation, edge computing, FPGA, neuromorphic computing, spiking neural networks

## Abstract

Spiking neural networks (SNNs) are a promising computational paradigm for resource- and energy-constrained edge applications. However, their implementation on field-programmable gate arrays (FPGAs) remains a complex task that typically requires both neuromorphic-computing knowledge and hardware-description expertise. This work presents ReSNNance, an open-source framework for the automatic generation of synthesizable VHDL implementations from high-level software descriptions of SNNs. ReSNNance provides a Python-based workflow in which users define the network architecture, neuron parameters, numerical precision and target configuration, while the framework generates the corresponding hardware modules, parameter files and interconnections. Its modular architecture supports sequential networks composed of Dense, Conv2D and pooling layers based on fixed-point leaky integrate-and-fire neurons. The generated VHDL is intended to remain readable and configurable, allowing users to inspect, modify and integrate the resulting circuits into larger hardware designs. ReSNNance also supports design-space exploration through configurable numerical formats and network parameters, facilitating the evaluation of trade-offs between classification accuracy, resource utilization and performance. The complete workflow was demonstrated using a large fully connected network and a spiking LeNet-5 architecture for MNIST classification. Both networks were automatically translated into VHDL, verified against their software counterparts and implemented on an AMD Zynq UltraScale+ FPGA. The results show that ReSNNance provides a practical path from software-defined SNN models to functional FPGA implementations by automating the generation of the corresponding VHDL modules and network interconnections.

## 1 Introduction

Artificial Neural Networks (ANNs) are widely used in applications involving large-scale data processing and machine learning, including image and speech recognition, autonomous systems, communications, and healthcare (Fourati et al., 2020; Chen et al., 2018). The increasing computational and energy requirements of these applications have encouraged the investigation of alternative processing paradigms, particularly for resource-constrained edge devices.

By representing information through discrete spike events and incorporating temporal dynamics into their neuron models, Spiking Neural Networks (SNNs) provide a computational model that is more closely related to biological neural processing than conventional ANNs. Their sparse activity can potentially be exploited by event-driven hardware to reduce unnecessary computation and data movement. This has motivated their use in applications involving temporal information, real-time inference, and energy-constrained processing (Ivanov et al., 2022; Schuman et al., 2022). However, the efficiency advantages associated with SNNs depend strongly on the network architecture, encoding method, workload, and implementation platform, and cannot be assumed for every application (Yamazaki et al., 2022).

Several challenges continue to limit the broader adoption of SNNs. Their methods for information encoding, network training, and model evaluation are less standardized than those used for conventional ANNs, and different applications may require substantially different neuron models and temporal representations (Davidson and Furber, 2021). Moreover, the potential reductions in energy consumption and hardware resources must be demonstrated using physical implementations and appropriate evaluation methodologies rather than inferred solely from the sparsity of spike activity.

Specialized digital hardware provides a way to exploit the computational characteristics of SNNs. Neuromorphic processors and configurable digital architectures can implement neuron dynamics, synaptic operations, and spike communication directly in hardware (Basu et al., 2022). In particular, Field Programmable Gate Arrays (FPGAs) provide configurable logic, arithmetic, and memory resources that support the exploration of different numerical representations and network architectures. Nevertheless, obtaining an efficient implementation generally requires making decisions about data representation, neuron and synapse organization, memory allocation, communication, and temporal execution. Translating a software-defined network into a synthesizable circuit therefore remains a specialized and potentially labor-intensive process.

A broad range of software libraries has been developed for the simulation and training of SNNs. Many are based on PyTorch and provide integration with machine-learning workflows (Hazan et al., 2018; Mozafari et al., 2019; Pehle and Pedersen, 2021; Eshraghian et al., 2023; Fang et al., 2023), while others use independent simulation environments or alternative software platforms (Rueckauer et al., 2017; Rasmussen, 2019; Snyder et al., 2023). These tools facilitate network development at the software level, but the transition from a trained model to a hardware implementation commonly requires a separate design flow. Differences between software neuron models, numerical representations, supported layers, and hardware execution semantics further complicate this translation.

Several frameworks have addressed parts of this software-to-hardware gap by providing compilation flows, hardware libraries, or automatic accelerator generation (Carpegna et al., 2022; Gerlinghoff et al., 2022). These approaches differ in their input representations, supported neuron and network models, synthesis methodologies, memory architectures, and dependence on vendor-specific tools. Consequently, there remains a need for accessible workflows that preserve a connection between the software model and the generated hardware while allowing the resulting implementation to be inspected and customized at the hardware-description level.

In this work, we present ReSNNance, an open-source framework for the automatic generation of synthesizable VHDL implementations from high-level descriptions of SNNs. As illustrated in Figure 1, ReSNNance acts as a bridge between software-level network development and digital hardware implementation. A network and its trained parameters are converted into an internal Model representation, from which the framework generates parameter files, hardware modules, and the interconnections required to construct a complete VHDL design.

**Figure 1:**
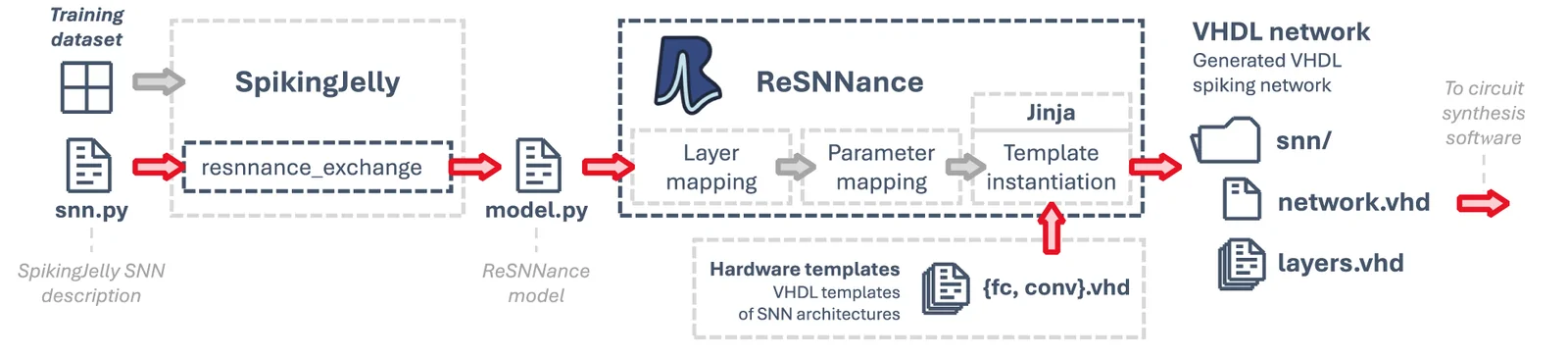
Overview of the ReSNNance software-to-hardware workflow. A high-level SNN is defined and trained using a PyTorch-based library and converted into a ReSNNance Model through a software exchange module. The framework maps this representation to parameterized hardware templates and generates the interconnected VHDL modules required to implement the complete network.

The current implementation focuses on sequential feed-forward networks composed of fully connected, convolutional, and pooling layers based on a simplified leaky integrate-and-fire neuron model. It supports configurable fixed-point representations and uses a template-based generation system that preserves access to the generated VHDL.

ReSNNance is distinguished by the combination of a SpikingJelly-compatible software interface, a hardware-equivalent neuron abstraction, and the automatic generation of readable and modifiable VHDL. Rather than optimizing a fixed accelerator architecture, the framework preserves a direct connection between the trained software model and the generated hardware description, including its topology, parameters, numerical representation, and neuron-update semantics.

The main contributions of this work are as follows:

- A high-level software-to-VHDL workflow: ReSNNance provides a Python-based interface for describing supported SNN architectures and automatically generates the corresponding parameterized and interconnected VHDL modules.
- Integration with software-defined SNN models: An exchange module converts networks developed using SpikingJelly into the internal ReSNNance representation, including their topology, neuron parameters, and previously trained weights.
- Template-based and configurable hardware generation: The framework uses reusable VHDL templates for fully connected, convolutional, pooling, and input-conversion layers. Numerical precision and network parameters can be configured before hardware generation, while the resulting source code remains available for inspection and modification.
- Software–hardware functional verification: The generated layers and complete networks can be evaluated using self-checking VHDL testbenches that compare their output spike trains with those produced by the corresponding software models.
- End-to-end evaluation on complete networks: The workflow is demonstrated using a spiking LeNet-5 architecture and a large fully connected network, covering different layer types, numerical precisions, and hardware-resource requirements.

Although the modular organization is intended to facilitate the incorporation of additional layers, neuron models, and hardware architectures, these extensions are not part of the functionality evaluated in this work. The two case studies were developed using SpikingJelly, converted into ReSNNance Models, and automatically translated into synthesizable VHDL. The LeNet-5 network demonstrates the integration of convolutional, pooling, and fully connected layers within a complete hardware architecture. The large fully connected network is used to examine how the generated implementation scales with network size and numerical precision within the resource limits of the selected FPGA. Both designs were functionally verified and implemented using the AMD Vivado design flow on a Zynq UltraScale+ MPSoC ZCU104 device. These experiments evaluate the applicability of the proposed workflow and illustrate the relationships between numerical precision, classification accuracy, resource utilization, operating frequency, and estimated power in the configurations considered.

The remainder of this paper is organized as follows. Section 2 describes the internal organization of ReSNNance, including its software abstractions and hardware templates. Section 3 presents the high-level interface and its integration with SpikingJelly. Section 4 describes the functional verification methodology and the two hardware case studies. Section 5 discusses the current scope of the framework, compares it with related FPGA-based SNN tools, and identifies its main limitations. Finally, Section 6 summarizes the conclusions of the work.

## 2 Framework

ReSNNance represents an SNN as a Python Model composed of an ordered sequence of Layer objects. Each supported layer is associated with a parameterized VHDL template. During compilation, the framework maps the network topology, trained weights, and additional parameters to these templates and generates the interconnected modules required to implement the complete network.

The framework can also generate build environments for synthesis with AMD Vivado and simulation with GHDL. VHDL source files are produced using the Jinja template engine (Pallets Projects, 2026), which substitutes network parameters and structural information into the corresponding hardware templates.

ReSNNance is open source and provides an extensible Application Programming Interface (API) through which additional hardware cores, layer types, connectivity schemes, and network architectures can be incorporated. The framework is distributed through the Python Package Index and its source code is available at github.com/resnnance/resnnance.

### 2.1 Models and layers

A ReSNNance Model represents an SNN and contains the information required to generate its hardware implementation.

As shown in the Unified Modeling Language (UML) diagram in Figure 2, Models are based on a list of all the network layers and a compiler in charge of mapping the abstract model into a standalone VHDL module. The Layer object is an abstract base class from which the supported spiking-layer implementations are derived. It is implemented by several instantiable layers:

- Dense: Represents a fully connected spiking layer. It is configured using the Leaky Integrate-and-Fire (LIF) decay parameter α and a two-dimensional NumPy weight array with dimensions (*N*_out_, *N*_in_).
- Conv2D: Represents a two-dimensional convolutional spiking layer. Its configuration includes the LIF decay parameter α, the input-feature-map dimensions, the kernel stride, and a four-dimensional NumPy weight array with dimensions (*K*_*y*_, *K*_*x*_, *C*_in_, *C*_out_).
- Pooling: Represents a two-dimensional average-pooling layer. Its configuration contains the input-feature-map dimensions and the pooling-window dimensions (*P*_*y*_, *P*_*x*_), together with the parameters required by the corresponding spiking-layer implementation.

**Figure 2:**
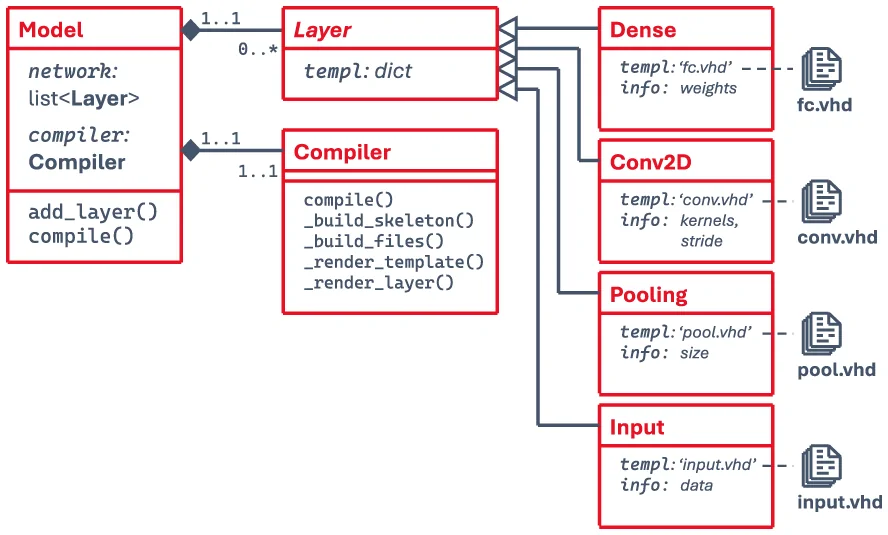
UML diagram of the class hierarchy in ReSNNance. A Model is constructed by sequentially adding Layer objects. Its associated Compiler maps each layer to the corresponding VHDL template and generates the complete hardware description.

### 2.2 Hardware templates

Each supported layer is linked to a parameterized VHDL template that is instantiated using the trained weights and structural information stored in the software model. The Compiler generates the individual layer modules and connects them sequentially using a network-level template, producing a standalone synthesizable SNN implementation.

The default template kit is based on the hardware architecture presented in López-Asunción and Ituero (2024). It provides implementations of the supported layers, together with the modules required for spike communication and simulation-time-step control. The current templates implement a simplified LIF neuron whose decay behavior is configured through the parameter α. The template-based organization is intended to facilitate the incorporation of additional neuron models, but these are not supported or evaluated in the present implementation.

### 2.3 Input conversion layer

The framework also includes a conversion layer for testing purposes, which instantiates an internal VHDL template that converts a non-spiking input vector (e.g. an image) into a spiking representation. It internally stores an 8-bit fixed-point real-valued input and generates a stream of spikes for each item to feed the synthesized SNN. This is done through discrete-time stochastic rate encoding, in which the probability of generating a spike at each simulation time step is proportional to the input pixel value.

For a constant input value, a homogeneous Poisson rate code can be approximated in discrete time by performing a Bernoulli trial at each simulation time step (Dayan and Abbott, 2001). These sequences of random spikes can be generated using a sequence of uniformly distributed random numbers between 0 and 1. For each timestep *n*, if the generated number *y*[*n*] is below a probability threshold *P* which is proportional to the desired spike rate *r* as


$$\begin{array}{l} y\left[n\right]<P\left(\text{spike in }\Delta t\right)=t_{step}r \end{array}$$ (1)

a spike *s*[*n*] = 1 is generated. The resulting discrete-time stochastic spike train has an expected firing rate *r* and approximates a homogeneous Poisson process as the simulation time step is reduced.

Let *v*∈[0, 1] be one of the original input values. We can define the firing rate as a function of *v* as *r* = *r*_*max*_*v* where *r*_*max*_ is the desired maximum rate. Then, the probability condition turns into


$$\begin{array}{l} y\left[n\right]<t_{step}r_{max}v \end{array}$$ (2)

For the hardware implementation, we assume the input values are encoded as *k*-bit long integer values *v*′∈[0, 2^*k*^−1] to map a specific input value *v* to a spiking rate *r*, so the scale factor is $s_{v}=2^{-k}$. Likewise, the random sequence is encoded as an *l*-bit long integer *y*′[*n*]∈[0, 2^*l*^−1], so its scale factor is $s_{y}=2^{-l}$. We can rewrite the condition as


$$\begin{array}{l} s_{y}y^{\prime }\left[n\right]<t_{step}r_{max}s_{v}v^{\prime } \end{array}$$

We can integrate all the constant multipliers into a single power-of-two constant as


$$\begin{array}{l} y^{\prime }\left[n\right]<2^{\lambda }v^{\prime } \end{array}$$ (3)

Equations 1–3 describe the successive transformation from the desired firing rate to the probability comparison implemented in the hardware domain. where λ can be found as a function of *r*_*max*_ and *t*_*step*_ as


$$\begin{array}{l} \lambda =\log_{2}\left(r_{max}t_{step}\frac{s_{v}}{s_{y}}\right) \end{array}$$ (4)

which can be tweaked to get an integer value and simplify the spiking condition as


$$s[n]=\%\{\begin{array}{ll} 1, & \text{if }y^{\prime }[n]<(v^{\prime }<<\text{λ})\\ 0, & \text{otherwise} \end{array}$$ (5)

We use a Linear Feedback Shift Register (LFSR) as a low-resource way to generate a pseudorandom signal (Dunn and Shultis, 2023). Taking the LFSR output value for *y*′[*n*], we can implement Equation 5.

As shown in Figure 3, the input values *v*′ are stored in RAM. When a simulation time step is triggered, the input layer circuit starts reading each input value out, shifting λ bits left and comparing them against the LFSR *y*′[*n*] value to generate an output spike *s*[*n*]. This process is repeated until the whole input vector is read, and starts over when the next time step tick arrives.

**Figure 3:**
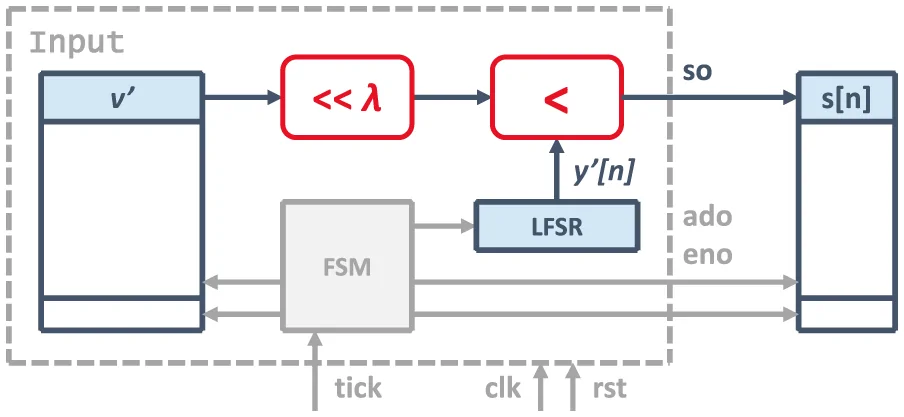
Schematic view of the Input layer. An internal memory holding the input vector values *v*′ is scanned to generate a stream of output spike vectors so using an LFSR pseudorandom number generator. The signals ado and eno can be used to store the output into an external memory.

## 3 High-level interface

SNNs intended for hardware inference are commonly trained using software frameworks and subsequently converted into a representation compatible with the target neuromorphic platform. SpikingJelly (Fang et al., 2023) provides a PyTorch-based environment for defining, training, and simulating SNNs, together with conversion mechanisms for deployment on supported neuromorphic platforms. Its software abstractions and integration with the PyTorch training workflow make it a suitable front-end for ReSNNance.

To connect both environments, we developed a SpikingJelly exchange module that converts trained networks into the internal ReSNNance Model representation. Training and software-level simulation are performed using SpikingJelly, whereas ReSNNance maps the resulting topology, neuron parameters, and quantized weights to the corresponding VHDL hardware templates. This separation allows the software training workflow to be retained while automating the generation of the hardware description used for inference.

The project also includes a legacy PyNN (Davison et al., 2009) front-end developed during the initial stages of ReSNNance. This interface remains available for converting supported PyNN network descriptions into the internal Model representation. However, current framework development and the experimental validation presented in this work focus on the SpikingJelly exchange module.

### 3.1 SpikingJelly interface

SpikingJelly extends PyTorch with stateful spiking-neuron modules, surrogate-gradient training functions, ANN-to-SNN conversion utilities, neuromorphic datasets, and GPU-accelerated simulation. Spiking networks are defined as subclasses of torch.nn.Module and constructed by composing synaptic layers with stateful neuron modules. This organization allows conventional PyTorch optimization procedures to be applied to networks containing temporal spiking dynamics.

The standard SpikingJelly LIF neuron is implemented by neuron.LIFNode. Its parameters include the membrane-potential time constant, reset potential, and surrogate function used for BPTT. SpikingJelly also provides exchange mechanisms for external neuromorphic environments. In particular, its lava_exchange module groups synaptic operations and spiking neurons into BlockContainer objects that can be converted into the Lava representation for execution on Loihi (Davies et al., 2018). SpikingJelly has also provided conversion support for the Lynxi KA200 platform (Deng et al., 2020).

A BlockContainer combines a synaptic operation, such as a linear, convolutional, or pooling layer, with a stateful spiking-neuron module. It behaves as a conventional PyTorch module during training while retaining the structural information required for subsequent conversion. This abstraction provides the basis for the ReSNNance exchange module.

### 3.2 ReSNNance exchange module

Following the container-based organization used by the SpikingJelly exchange mechanisms, we developed the resnnance_exchange module shown in Figure 4. It introduces a hardware-equivalent neuron model, RsnnNode, and a corresponding BlockContainer. Together, these components preserve the layer organization required by the ReSNNance conversion process while allowing the network to be trained and simulated within SpikingJelly.

**Figure 4:**
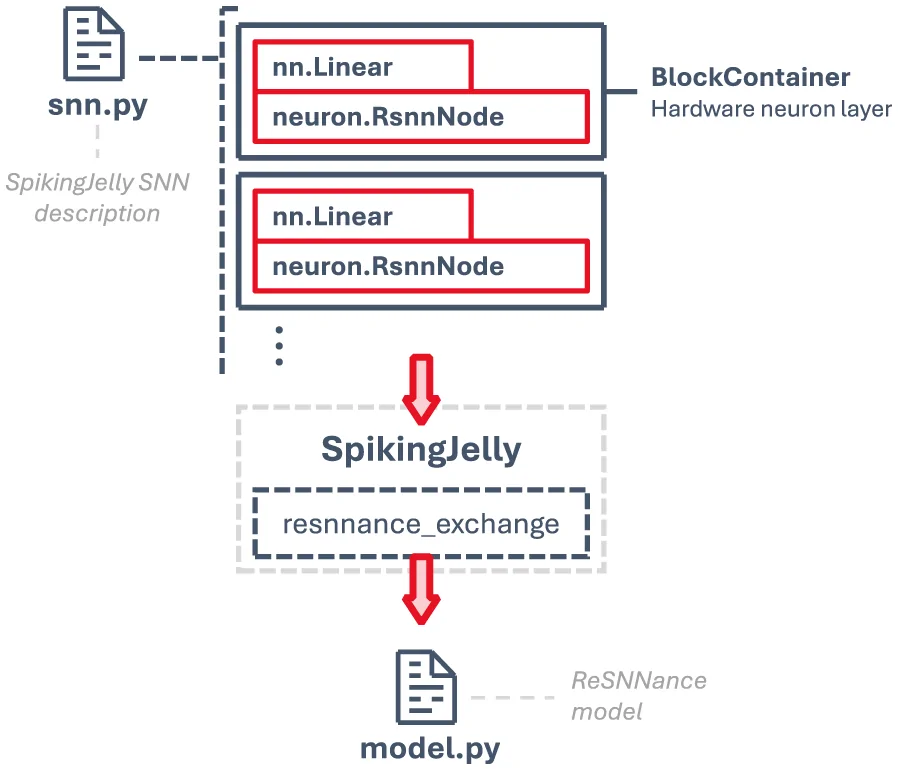
Conversion of a SpikingJelly SNN into a ReSNNance Model using the resnnance_exchange module. Synaptic layers and RsnnNode instances are grouped into BlockContainer objects, from which the corresponding ReSNNance layers are generated.

#### 3.2.1 RsnnNode

The standard SpikingJelly neuron.LIFNode does not directly reproduce the update order and parameterization of the simplified LIF neuron implemented by the default ReSNNance hardware templates. The relevant differences are:

- SpikingJelly expresses the decay using a real-valued time constant τ, whereas the default ReSNNance hardware represents it using an integer parameter α, with τ = 2^α^.
- The hardware model distinguishes between the resting potential *u*_rest_ and the reset potential *u*_reset_.
- The order in which synaptic integration and membrane-potential decay are applied differs from the standard neuron.LIFNode update.

In the standard LIF formulation, the subthreshold membrane dynamics can be written as


$$\begin{array}{l} \frac{du}{dt}=-\frac{1}{\tau }\left(u-u_{\text{reset}}\right). \end{array}$$ (6)

Let


$$\begin{array}{l} x\left[n\right]=\underset{i}{\sum }\omega_{i}s_{i}\left[n\right] \end{array}$$ (7)

Equations 6 and 7 define the continuous-time LIF dynamics and the corresponding discrete synaptic input used in the subsequent formulations. denote the synaptic input at time step *n*, where ω_*i*_ and *s*_*i*_[*n*] are the *i*th synaptic weight and input spike, respectively. SpikingJelly provides two discrete charge formulations, depending on whether the synaptic input is affected by the decay factor:

When the synaptic input is affected by the decay factor,


$$\begin{array}{l} u\left[n+1\right]=u\left[n\right]+\frac{1}{\tau }\left[x\left[n\right]-\left(u\left[n\right]-u_{\text{reset}}\right)\right] \end{array}$$ (8)

$$\begin{array}{l} u\left[n+1\right]=u\left[n\right]+x\left[n\right]-\frac{1}{\tau }\left(u\left[n\right]-u_{\text{reset}}\right) \end{array}$$ (9)

In the ReSNNance hardware neuron, the synaptic contribution is accumulated before the decay term is applied. Defining the post-integration intermediate potential as


$$\begin{array}{l} u_{g}\left[n\right]=u\left[n\right]+x\left[n\right], \end{array}$$ (10)

the hardware update becomes


$$\begin{array}{l} u\left[n+1\right]=u_{g}\left[n\right]-\frac{1}{\tau }\left(u_{g}\left[n\right]-u_{\text{rest}}\right). \end{array}$$ (11)

For the configuration *u*_rest_ = *u*_reset_, this expression can be written as


$$\begin{array}{l} u\left[n+1\right]=u\left[n\right]+x\left[n\right]-\frac{1}{\tau }\left(u\left[n\right]+x\left[n\right]-u_{\text{reset}}\right). \end{array}$$ (12)

Equations 10–12 define the intermediate integration step and the resulting hardware-compatible membrane-potential update. This update is not equivalent to either of the standard formulations in Equations 8 and 9, because the decay term is evaluated after the synaptic contribution has been added. Although the numerical difference may be small for some parameter and input configurations, reproducing the update order is necessary for software–hardware functional equivalence.

The RsnnNode class therefore inherits from neuron.BaseNode and implements the hardware-compatible update through its neuronal_charge() function. The integer parameter α is used to represent the decay factor, ensuring that the software model follows the same numerical update employed by the generated VHDL neuron.

The same approach could be extended to other hardware neuron models by implementing corresponding SpikingJelly nodes that reproduce their update equations. Such extensions, however, are not included or evaluated in the current version of the framework.

#### 3.2.2 BlockContainer

The ReSNNance-compatible BlockContainer combines a supported synaptic layer with an RsnnNode. Each container implements a to_resnnance() conversion function that generates the corresponding layer in the ReSNNance framework representation. Applying this conversion sequentially to a network composed of supported containers produces a complete ReSNNance Model containing the trained weights, neuron parameters, and layer-connectivity information.

For networks receiving static non-spiking data, the conversion process can optionally prepend the input-conversion layer described in Section 2.3. This layer performs the hardware rate encoding required to transform the input values into spike trains before they are processed by the converted network.

## 4 Testing and validation

To demonstrate the applicability of the ReSNNance workflow and exercise the supported hardware components, two representative case studies were selected:

- A LeNet-5 convolutional SNN. This network demonstrates the implementation and seamless integration of the fully connected, convolutional, and pooling hardware modules generated by ReSNNance into a complete network architecture.
- A large fully connected SNN. This architecture examines how the generated hardware scales with network size and numerical precision within the resource limits of the selected FPGA.

Both networks were developed in Python using the SpikingJelly library and exported through the resnnance_exchange module. These models were then compiled by the ReSNNance toolchain to generate synthesizable VHDL descriptions, which were implemented using Vivado Design Suite 2022.2 on the AMD ZCU104 evaluation board, featuring a Zynq UltraScale+ MPSoC ZU7EV device.

All implementations include the input conversion layer using 8-bit input image values ($s_{v}=2^{-8}$), the membrane potential is represented through 16-bit signed fixed-point variables, and stochastic spike generation is performed using a 16-bit LFSR ($s_{y}=2^{-16}$). With *r*_*max*_ = 1/*t*_*step*_, this results in λ = 8 according to Equation 4.

The reported results were obtained at a clock frequency of 100 MHz. Power was estimated assuming an ambient temperature of 25 ° using the Vivado power analysis flow, with Switching Activity Interchange Format (SAIF) activity generated during post-implementation timing simulation. The values correspond to the estimated average device power over the simulated inference interval and include static, clock, signal, and logic contributions. They should therefore be interpreted as tool-based estimates rather than direct board-level measurements.

The networks were trained on the standard 60,000-sample MNIST training partition and evaluated on the corresponding 10,000-sample test partition. Training was performed in SpikingJelly using BPTT with the arctangent surrogate function over 25 simulation time steps. The LeNet-5 and large fully connected architectures are described in Sections 4.2 and 4.3, respectively. The neuron threshold, reset potential, and decay parameter were set to *v*_*th*_ = 1, *v*_*reset*_ = 0, and 1/τ = 0.25 (τ = 4), respectively, using zero reset.

After training, weights were quantized to signed 8-, 6-, and 4-bit representations using global scaling. Values were rounded to the nearest quantization level using round-half-to-even rounding and saturated to the representable range. Membrane potentials were represented using 16-bit signed fixed-point variables. The accuracy reported in Tables 1, 2 was obtained from quantized SpikingJelly inference on the MNIST test set.

**Table 1:** Hardware metrics on ZCU104 and performance for the LeNet-5 convolutional SNN.

Weight bits	LUTs	FFs	BRAM	DSP	Max. Freq.	Accuracy
8	19,282 (8.37%)	19,542 (4.24%)	0	0	290.5 MHz	99.06%
6	17,012 (7.38%)	17,200 (3.73%)	0	0	255.6 MHz	98.86%
4	12,466 (5.41%)	14,437 (3.13%)	0	0	356.9 MHz	98.17%

**Table 2:** Hardware metrics on ZCU104 and performance for the large fully connected SNN.

Weight bits	LUTs	FFs	BRAM	DSP	Max. Freq.	Accuracy
8	203,907 (88.50%)	76,288 (16.56%)	0	0	161.5 MHz	98.52%
6	160,464 (69.65%)	71,598 (15.54%)	0	0	157.04 MHz	98.46%
4	103,795 (45.05%)	65,337 (14.18%)	0	0	192.4 MHz	98.18%

The generated network operates as a pipeline in which all layers process each simulation time step concurrently. Consequently, the minimum interval between consecutive input ticks is determined by the layer with the longest processing time. Injecting a new tick before this layer has consumed the data associated with the previous one could overwrite data that are still being processed. The cycle-level timing model of the fully connected, convolutional, and pooling architectures is described in detail in López-Asunción and Ituero (2024).

Building on this setup, the proposed toolchain was evaluated through an exploratory assessment of the resulting implementations in terms of hardware resource utilization, maximum operating frequency, and power consumption. To further assess the flexibility of the generated hardware, these metrics were examined across three weight quantization levels: 8, 6, and 4 bits.

The two case studies were selected to exercise complementary parts of the supported framework. LeNet-5 combines convolutional, pooling, and fully connected layers, whereas the large fully connected network stresses distributed parameter storage and FPGA resource utilization.

### 4.1 Functional verification

Before synthesis and implementation, the generated hardware was functionally verified through VHDL simulation using self-checking testbenches. Each supported layer type was evaluated using representative 32 × 32 and 1024 × 1024 configurations. The Conv2D tests additionally covered 3 × 3 and 5 × 5 kernels. The evaluated numerical formats used 4-, 6-, 8-, and 16-bit-wide integer values. Deterministic input spike trains and quantized parameters were applied to both the ReSNNance software model and the generated VHDL implementation, and their output spike trains were compared cycle by cycle over 25 simulation time steps. These tests were intended to cover representative layer sizes, convolutional kernels, and numerical formats rather than every possible parameter combination.

Complete generated networks were subsequently simulated using the same input and parameter files employed during synthesis, thereby verifying the consistency of the generated layer interconnections and network-level execution. No mismatches were observed between the software and VHDL output spike trains for the evaluated configurations.

### 4.2 LeNet-5 convolutional network

The first case study is based on the LeNet-5 spiking neural network shown in Figure 5. The network was developed in SpikingJelly and trained on the non-spiking MNIST dataset using Backpropagation Through Time (BPTT).

**Figure 5:**
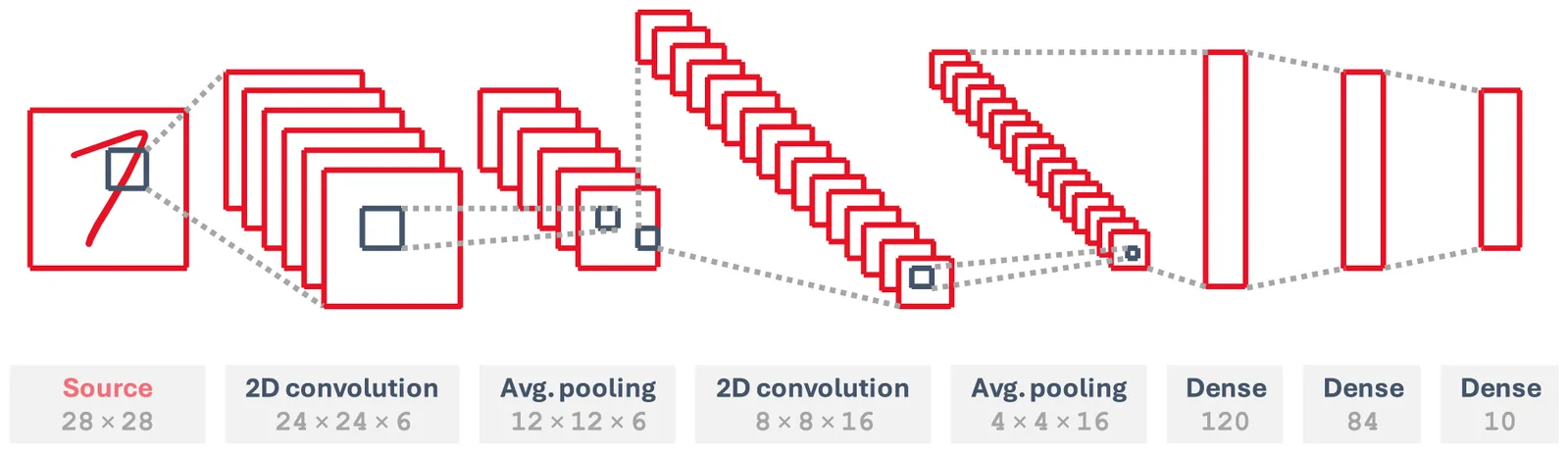
Spiking LeNet-5 architecture for a 28 × 28 MNIST input image and 10 output classes. The labels indicate the output dimensions of each layer. For example, the first convolutional layer applies six kernels and produces six 24 × 24 feature maps.

Table 1 summarizes the hardware resource utilization, maximum operating frequency, and classification accuracy obtained for the three weight quantization levels considered.

The results show that hardware resource utilization improves as weight precision decreases, while classification accuracy is progressively degraded. Relative to the 8-bit implementation, reducing the weight precision to 6 bits decreases Look-Up Table (LUT) and Flip-Flop (FF) utilization by 11.8% and 12.0%, respectively, with only a 0.20 percentage-point reduction in classification accuracy. However, the maximum operating frequency is reduced from 290.5 MHz to 255.6 MHz. Further reducing the precision to 4 bits lowers LUT and FF utilization by approximately 35.4% and 26.1%, respectively, and increases the maximum operating frequency by 22.9% at the cost of a 0.89 percentage-point reduction in accuracy, with none of the evaluated configurations requiring Digital Signal Processing (DSP) or Block RAM (BRAM) resources. This is consistent with the event-driven nature of the underlying SNN hardware architecture, in which synaptic contributions are accumulated upon spike arrival rather than computed through multiply-accumulate operations, thereby eliminating the need for dedicated DSP resources; similarly, the default template kit maps weights and neuron states to LUT- and register-based resources rather than to dedicated block memories.

SAIF activity was generated from post-implementation simulation using MNIST input samples rather than random switching assumptions. The same samples were used for all weight-precision configurations, and switching activity was accumulated over the complete 25-time-step inference interval. As described at the beginning of Section 4, the minimum interval between consecutive input ticks is determined by the layer with the longest processing time. For the implemented LeNet-5 architecture, this critical stage corresponds to the second convolutional layer. This layer receives six input feature maps of size (12 × 12) and applies (5 × 5) kernels to produce (16) output feature maps of size (8 × 8). Operating at a clock frequency of 100 MHz, this results in a minimum interval of 139.78 μ s between consecutive time steps.

Post-implementation timing simulation was subsequently used to measure an end-to-end latency of 320.98 μ s from the injection of the first input tick to the availability of the corresponding network output. Once the pipeline is filled, subsequent outputs are produced at intervals of the tick period. The complete inference over 25 simulation time steps therefore requires 3675.7 μ s.

Figure 6 summarizes the estimated static and dynamic power consumption for the evaluated weight quantization levels. These results indicate that, although the device static power sets the dominant contribution to the overall consumption, the proposed hardware generation framework enables reducing the dynamic power through weight quantization, consistently with the reduction in hardware resources reported in the previous section.

**Figure 6:**
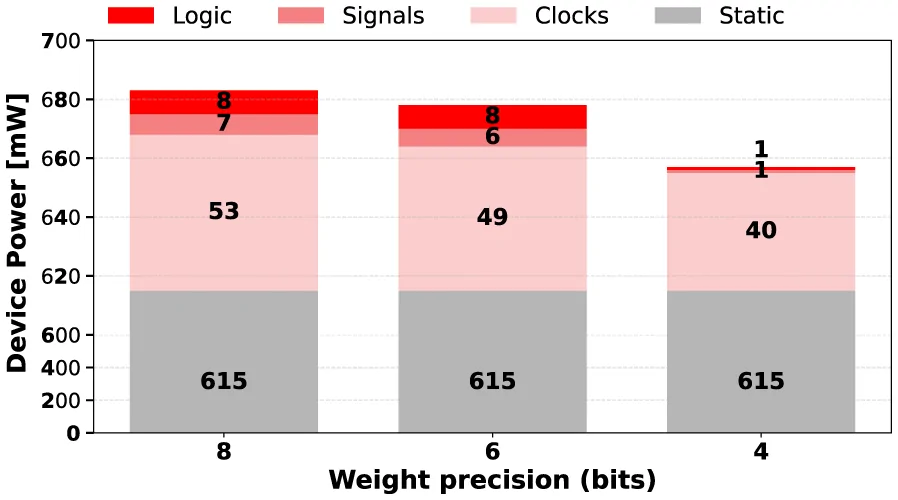
Estimated device power for the LeNet-5 implementations with 8-, 6-, and 4-bit weights at a 100 MHz operating frequency and an ambient temperature of 25°C. Power components were obtained from post-implementation analysis using SAIF activity generated over a complete 25-time-step inference simulation.

### 4.3 Large fully connected network

To examine the scaling behavior of the generated hardware under high resource utilization, a large fully connected SNN was implemented on the selected FPGA. This case study evaluates how network size and numerical precision affect resource utilization and implementation performance within the capacity of the target device.

As the input layer contains 784 features, corresponding to the 28 × 28 input image, the network topology was defined using hidden layers of the same dimensionality, with an output layer of 10 neurons matching the number of MNIST classes. Additional hidden layers were incorporated progressively until the available FPGA resources were exhausted. Using this methodology, the largest successfully implemented architecture reached a total of 1,237,152 weights (784-784-784-10), enabling the analysis of the resource limits and performance characteristics of the proposed mapping approach on the selected platform.

Table 2 reports the hardware resource utilization, maximum operating frequency, and classification accuracy of the large fully connected implementation for the three weight quantization levels considered, where LUT utilization reaches 88.50% at 8-bit precision, close to the practical capacity of the target device. Relative to the 8-bit implementation, reducing the weight precision to 6 bits decreases LUT and FF utilization by 21.3% and 6.2%, respectively, with a marginal 0.06 percentage-point reduction in classification accuracy, while the maximum operating frequency remains nearly unchanged, decreasing slightly from 161.5 MHz to 157.04 MHz. Further reducing the precision to 4 bits lowers LUT and FF utilization by 49.1% and 14.4%, respectively, and increases the maximum operating frequency to 192.4 MHz, at the cost of a 0.34 percentage-point reduction in accuracy. Given the high LUT utilization observed across all configurations, Table 3 presents a detailed breakdown of LUT usage into logic, distributed RAM, and shift register components to further analyze how these resources are distributed.

**Table 3:** LUT utilization breakdown.

LUTs	LUTs as logic	LUTs as distributed RAM	LUTs as shift register
203,907	203,180	682	45
160,464	159,737	682	45
103,795	102,862	682	251

As can be observed, the vast majority of LUTs are used as logic across all configurations, consistently with the overall decreasing trend observed in resource utilization. The number of LUTs used as distributed RAM remains constant at 682 across all three quantization levels, indicating that this resource is independent of weight bit-width. In contrast, LUTs used as shift registers remain constant at 45 for the 8-bit and 6-bit configurations but increase to 251 at 4-bit precision.

In addition to resource utilization, power consumption was estimated using the methodology described at the beginning of Section 4. For this network, the tick period is determined by the largest layer, which requires 784 clock cycles to write its input values and 784 clock cycles to produce its output, resulting in a minimum inter-tick period of 15.72 μs at a clock frequency of 100 MHz. Post-implementation timing simulation measured an end-to-end latency of 39.42 μs for a single simulation time step, from the injection of the input tick to the availability of the corresponding network output. Consequently, the complete inference over 25 simulation time steps requires 416.7μs.

As shown in Figure 7, static power remains nearly constant across all configurations (618–622 mW), as expected given its dependence on the device rather than on the implemented logic. In contrast, dynamic power components decrease consistently with weight precision, following the same trend observed in hardware resource utilization. The logic power drops from 750 mW at 8-bit precision to 567 mW at 6-bit precision and further to 307 mW at 4-bit precision, with signal and clock power exhibiting a similar decreasing trend. Unlike the LeNet-5 case study, dynamic power dominates the overall consumption here, consistently with the substantially higher resource utilization required by this network.

**Figure 7:**
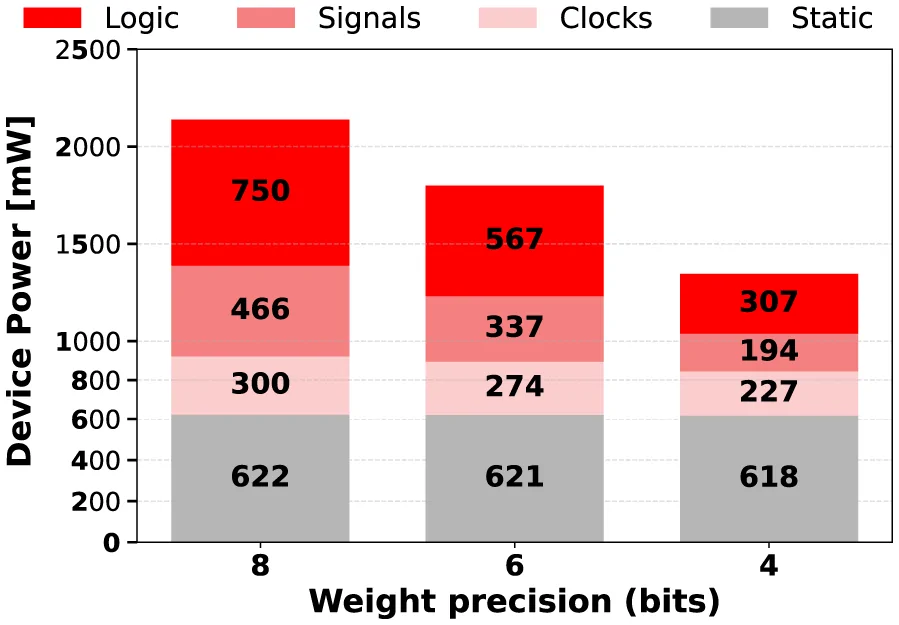
Estimated device power for the Large Fully Connected implementations with 8-, 6-, and 4-bit weights at a 100 MHz operating frequency and an ambient temperature of 25°C. Power components were obtained from post-implementation analysis using SAIF activity generated over a complete 25-time-step inference simulation.

## 5 Discussion

The results presented in the previous section demonstrate the feasibility of using ReSNNance to translate software-defined spiking neural networks into synthesizable hardware descriptions. Beyond the performance of the individual case studies, the main contribution of the framework lies in the automation and integration of the different stages of this workflow. This section discusses the current scope of ReSNNance, its position relative to other FPGA-oriented SNN frameworks, and the limitations that define the applicability of the present results.

### 5.1 Scope of the current framework

ReSNNance is intended to bridge the gap between software-level SNN development and hardware implementation. Users specify the network structure, neuron parameters, numerical representation, and implementation configuration through a Python interface. From this description, the framework generates the corresponding parameter files, interconnected hardware modules, and synthesizable VHDL implementation.

The current version supports sequential feed-forward architectures composed of Dense, Conv2D, and pooling layers based on fixed-point leaky integrate-and-fire neurons. The generated description remains accessible at the VHDL level, allowing designers to inspect, modify, or integrate the resulting modules into larger hardware systems. Table 4 summarizes the current capabilities of the framework and distinguishes them from the subset that has been experimentally evaluated in this work.

**Table 4:** Current scope of ReSNNance and validation covered in this work.

Aspect	Current framework support	Validation presented in this work
Network topology	Sequential feed-forward architectures	Fully connected and convolutional feed-forward networks
Layer types	Input, dense, Conv2D, and pooling layers	Representative layer configurations and complete networks
Neuron model	Fixed-point leaky integrate-and-fire neurons	Software–hardware comparison of output spike trains
Numerical representation	Configurable fixed-point formats for parameters, weights, and internal signals	Multiple numerical precisions evaluated in the case studies
Model preparation	Software-side network definition and import of previously trained parameters	MNIST models prepared using the software workflow described in this work
Hardware generation	Parameterized VHDL modules, parameter files, and network interconnections	Synthesis and implementation using the AMD Vivado design flow
Target platform	Synthesizable VHDL intended for FPGA implementation	AMD Zynq UltraScale+ MPSoC ZCU104 evaluation board
Verification	Layer- and network-level VHDL simulation	Self-checking comparison between software and VHDL output spike trains
Software front-ends	Legacy PyNN interface and SpikingJelly exchange module	SpikingJelly exchange module

The distinction between supported functionality and experimental validation is important. The framework generates parameterizable VHDL rather than a hardware description tied to a single network instance. Nevertheless, the results reported here establish correct operation only for the layer configurations, network architectures, FPGA device, and synthesis flow included in the evaluation. Broader applicability should therefore be assessed using additional models and hardware targets.

### 5.2 Comparison with existing FPGA-based SNN frameworks

To position ReSNNance within the current landscape of FPGA-based SNN design tools, Table 5 presents a qualitative comparison against four representative FPGA-based SNN frameworks: NeuroFlow (Cheung et al., 2016), SyncNN (Panchapakesan et al., 2021), E^3^NE (Gerlinghoff et al., 2022), and Spiker+ (Carpegna et al., 2025). The comparison covers the main design dimensions relevant to hardware SNN generation, including input abstraction, synthesis toolchain, spike encoding, temporal execution model, supported neuron models, degree of hardware automation, target platform, numerical precision, memory architecture, power consumption, and open-source availability.

**Table 5:** Qualitative comparison of representative FPGA-based SNN frameworks.

Aspect	NeuroFlow	SyncNN	E^3^NE	Spiker+	ReSNNance
Problem solving	Biologically realistic SNN simulation.	Efficient deployment of CNN-to-SNN models on FPGAs.	Automatic generation of SNN hardware accelerators.	Simplified design of custom SNN accelerators.	Automatic conversion of trained models into VHDL accelerators.
Programming/input abstraction	PyNN (Python, simulator-independent).	Pre-trained CNN model, manually mapped to HLS layer templates.	PyTorch model + design variables (parallelism, resolution).	Python dictionary on top of snntorch.	SpikingJelly exchange module + PyNN interface (legacy).
Synthesis toolchain	Java (Maxeler MaxCompiler).	HLS C/C++.	Hand-crafted RTL/HDL block library.	Auto-generated VHDL from Python.	Jinja2 generating plain VHDL.
Spike encoding	From neuron ODE dynamics, no fixed scheme	Rate encoding (Poisson).	Radix encoding: binary-weighted spike positions (powers of two).	Rate encoding (Poisson) or pre-encoded external input.	Discrete-time stochastic rate encoding via an on-chip LFSR.
Temporal execution model	Time-driven, continuous simulation, 1 ms steps.	Synchronous: only the input layer runs multiple timesteps; the rest of the network is computed once per layer.	Sequential, layer-by-layer; each layer processes the full (compressed) T-step spike train before moving to the next.	Clock-driven, synchronous over N_cycles steps; layers operate concurrently; idle cycles skipped when no spikes are present.	Clock-driven, deeply pipelined; one simulation tick per timestep across all neuron cores.
Neuron models	LIF, adaptive exponential IF, Izhikevich, Hodgkin-Huxley.	IF only.	IF only.	IF, I-order LIF, II-order LIF (hard or subtractive reset).	Simplified LIF only (extendable).
Hardware automation degree	Complete—automatically derives parallelism, FPGA allocation, weight caching, and synaptic organization.	Partial—on-chip buffering automated; parallelism set manually per layer.	Complete—compiler auto-derives processing modules, memory sizing and instructions.	Complete—Python compiled to VHDL accelerator.	Complete—SpikingJelly compiled into VHDL build environment.
Target platform	Maxeler FPGA systems.	AMD SoCs.	AMD UltraScale+ FPGAs.	Low-end AMD FPGAs.	AMD UltraScale+ FPGAs.
Numerical precision	32/64-bit floating point + 16-bit weights.	16/8/4-bit fixed-point.	Configurable fixed-point.	Configurable fixed-point.	Configurable fixed-point.
Memory architecture	External DRAM + on-chip BRAM.	Hierarchical BRAM buffering.	BRAM/DRAM hybrid per layer.	BRAM + LUT/FF + DRAM fallback.	Distributed memory only.
Reported power	30–40 W.	0.4 W.	1.2 W.	180–430 mW.	0.66–0.68 W (LeNet-5); 1.35–2.14 W (large FC).
Open source	No	Yes	Yes	Yes	Yes

Reported hardware and power results correspond to different devices, networks, and evaluation methodologies and are therefore not directly comparable.

A direct quantitative ranking of these frameworks would not be methodologically reliable because the reported results use different network architectures, datasets, numerical precisions, FPGA devices, operating frequencies, and power-characterization procedures. The comparison is therefore intended to identify differences in functionality and design methodology rather than to establish performance superiority.

As shown in Table 5, all frameworks achieve a complete or partial degree of hardware automation, reflecting a broader trend toward automated SNN-to-hardware compilation flows. Among these, ReSNNance and Spiker+ share the closest design philosophy, both compiling models directly from a Python-based front-end into synthesizable VHDL without relying on High-Level Synthesis (HLS) or hand-crafted RTL libraries, unlike SyncNN and E^3^NE, which respectively depend on HLS C/C++ and a hand-crafted RTL/HDL block library.

In terms of spike encoding, ReSNNance adopts a discrete-time stochastic rate-encoding scheme similar to SyncNN and Spiker+, implemented on-chip through a pseudorandom LFSR generator, whereas E^3^NE relies on a fundamentally different radix-based binary-weighted encoding, and NeuroFlow derives spike timing directly from the underlying neuron ordinary differential equation (ODE) dynamics rather than from a fixed encoding scheme.

Among the frameworks considered in Table 5, ReSNNance is the only one whose evaluated implementations rely exclusively on distributed memory, in contrast to the BRAM-based or hybrid BRAM/Dynamic Memory (DRAM) architectures adopted by the remaining frameworks. This is consistent with the results discussed in the previous case studies, where no BRAM or DSP resources were required across all evaluated configurations.

The estimated device power obtained for the evaluated ReSNNance implementations falls within the sub-watt to low-watt range reported by several FPGA-based SNN frameworks. However, these values should not be interpreted as a direct power ranking. The evaluated networks, FPGA devices, operating frequencies, environmental conditions, activity generation procedures, and power characterization methodologies differ substantially across studies.

### 5.3 Limitations and future extensions

The present implementation supports a restricted subset of possible SNN architectures. It is currently focused on sequential feed-forward networks constructed from Dense, Conv2D, and pooling layers and using a simplified leaky integrate-and-fire neuron model. Recurrent connections, alternative neuron and spike-encoding models, online learning, and dynamic runtime reconfiguration are not addressed in the current version. Furthermore, the experimental evaluation is limited to rate-coded MNIST classification. Additional datasets and application domains will be required to assess the framework under more complex and heterogeneous workloads.

Addressing these architectural limitations would require extensions to different components of the framework. Supporting residual or branched networks would require extending the internal connectivity representation and network-level VHDL generator beyond sequential layer composition. Recurrent architectures would additionally require explicit feedback paths and scheduling mechanisms, while alternative neuron models would require corresponding software nodes and hardware templates implementing equivalent update equations.

The hardware evaluation also has a limited scope. Although ReSNNance generates synthesizable VHDL intended to reduce dependence on a particular implementation platform, all the results reported in this work were obtained using a single FPGA device and the AMD Vivado toolchain. Compatibility with other FPGA vendors and synthesis tools should therefore be regarded as a design objective rather than as an experimentally demonstrated property.

The generated architectures use distributed memory resources for the evaluated network parameters and do not require block RAM or DSP blocks in the configurations considered in this work. This design makes the arithmetic structure explicit, but it can also produce increasing LUT and routing requirements as network size grows. The large fully connected case study demonstrates that the framework can generate and synthesize a design containing more than one million weights, but it does not establish unlimited scalability. The practical network size remains constrained by the resources, memory organization, routing capacity, and timing characteristics of the target device.

Future development will consider additional neuron models, layer types, coding schemes, and network topologies, together with alternative memory organizations for larger networks. Validation using further datasets, FPGA families, and synthesis toolchains will also be necessary to evaluate portability more comprehensively. These extensions can be incorporated into the modular generation workflow without changing the main software-to-VHDL approach introduced in this work.

## 6 Conclusions

This work presented ReSNNance, an open-source framework for the automatic generation of synthesizable VHDL implementations from high-level descriptions of spiking neural networks (SNNs). The framework connects software-level network development with hardware implementation through a Python-based workflow that generates parameterized VHDL modules, parameter files, and the interconnections required to construct a complete network. Its template-based architecture currently supports sequential networks composed of fully connected, convolutional, and pooling layers using configurable fixed-point representations and a simplified leaky integrate-and-fire neuron model.

The complete workflow was evaluated using two MNIST classification networks developed with SpikingJelly. The LeNet-5 case study demonstrated the automatic integration of convolutional, pooling, and fully connected layers into a complete hardware implementation. The evaluated configurations achieved classification accuracies of up to 99.06%, while the 8-bit implementation reached a maximum operating frequency of approximately 290 MHz. The large fully connected case study demonstrated the generation and synthesis of a network containing 1,237,152 weights. Its largest evaluated configuration occupied 88.50% of the available LUT resources while maintaining a classification accuracy above 98% across the tested numerical precisions.

The results also showed how numerical precision affects classification accuracy, resource utilization, operating frequency, and estimated power within the evaluated architectures. None of these implementations required BRAM or DSP resources, as network parameters and arithmetic operations were mapped to distributed logic. This implementation strategy enables explicit and configurable hardware generation, although the increasing LUT and routing requirements observed for the large fully connected network constrain the size of the designs that can be mapped to a given device.

Overall, the results demonstrate that ReSNNance provides a functional software-to-VHDL workflow for the supported network architectures and facilitates the exploration of fixed-point SNN implementations. The generated descriptions remain accessible at the VHDL level, allowing hardware designers to inspect, modify, and integrate them into larger systems. Although the present evaluation was conducted using a single FPGA family and synthesis toolchain, the modular organization of the framework provides a basis for incorporating additional hardware templates, neuron models, layer types, memory architectures, and target platforms in future versions.

## Data Availability

The source code associated with this work is publicly available at https://github.com/resnnance/resnnance. The experiments reported in this article correspond to ReSNNance release v1.0 (commit b23d8a3). ReSNNance is distributed under the GPLv3 license. The MNIST dataset analyzed in this study is publicly available at https://yann.lecun.com/exdb/mnist/. No new datasets were generated as part of this work.
